# Polymerase chain reaction-based detection of coinfecting DNA viruses in Vietnamese pigs in 2017 and 2021

**DOI:** 10.14202/vetworld.2022.2491-2498

**Published:** 2022-10-29

**Authors:** Van Giap Nguyen, Huu Anh Dang, Thanh Trung Nguyen, Thi My Le Huynh, Ba Hien Nguyen, Le Anh Minh Pham, Huynh Thanh Phuong Le

**Affiliations:** 1 Department of Veterinary Microbiology and Infectious Diseases, Faculty of Veterinary Medicine, Vietnam National University of Agriculture, Hanoi, Vietnam; 2 Department of Pharmacology, Toxicology, Internal Medicine and Diagnostics, Faculty of Veterinary Medicine, Vietnam National University of Agriculture, Hanoi, Vietnam; 3 Department of Microbiology Technology, Faculty of Biotechnology, Vietnam National University of Agriculture, Hanoi, Vietnam; 4 Department of the Science and Technology, Vietnam National University of Agriculture, Hanoi, Vietnam

**Keywords:** co-infection, porcine bocavirus, porcine parvovirus, torque teno virus, Vietnamese pigs

## Abstract

**Background and Aim:**

Many studies have reported on the phenomenon of co-infections involving two or more pathogens (bacteria or viruses) over the past few years. However, very few studies on this issue were conducted in Vietnam. Therefore, this study aimed to determine the circulation of single and multiple porcine parvovirus (PPV) (e.g., PPV1, PPV2, PPV3, and PPV4), porcine bocavirus (PBoV), and torque teno virus (TTV) (TTV1 and TTV2) infections in Vietnamese pigs.

**Materials and Methods:**

A total of 174 porcine circovirus 2-positive samples from pigs (n = 86 for 2017 and n = 88 for 2021), including from the sera and internal organs, across 11 provinces were examined by polymerase chain reaction.

**Results:**

This study demonstrated the wide distribution of DNA viruses among pig farms in Vietnam in 2021, with the detection rate for PPV ranging from 3.4% to 27.3% among PPV1-PPV4. Moreover, the detection rates of TTV genotypes were confirmed to be 14.8% (TTV1) and 63.6% (TTV2), respectively, and the positive rate of PBoV was 65.9%. The most frequent combinations were double and triple infections. Double infection was found in 16/86 (18.6%) in 2017 and 26/88 (29.5%) in 2021, while triple infection was found at 19/86 (22.1%) in 2017 and 26/88 (29.5%) in 2021. The incidence of simultaneous detection of more than three viruses was low.

**Conclusion:**

These results provide at least partial information about the occurrence of three viruses, including PPV (including PPV1 to 4), PBoV, and TTV (TTV1 and TTV2), in pigs. Determination of particular viruses in pigs will help to prevent the porcine respiratory disease complex caused by DNA viruses in Vietnamese pigs in the future.

## Introduction

At present, next-generation sequencing has evolutionized almost all fields of biological science for research and diagnostic applications due to its high speed and throughput data generation [1, 2]. Specifically, it is commonly used in the diagnosis and effective discovery of novel RNA and DNA viruses and other pathogens [3]. Among those groups, single-stranded DNA (ssDNA) has been identified to have linear or circular genomes forms and has the potential ability to produce disease in conjunction with other pathogens [4]. However, pig-associated ssDNA viruses include porcine parvovirus (PPV) and porcine bocavirus (PBoV), both in the family of *Parvoviridae*, and torque teno virus (TTV) in the *Anelloviridae* family; their ability to causes diseases and to be pathogenic to pigs is still under debate [4, 5]. Thus, it is important to elucidate the association of these ssDNA viruses with diseases in pigs.

PPV is a small non-enveloped DNA virus considered to be one of the major causes of reproductive failure in swine worldwide [6]. To date, several novel parvoviruses have been reported in pigs, namely PPV2–PPV7. Contradictory to PPV1, which belongs to the genus *Protoparvovirus*, the emerging PPV species belong to the genera *Tetraparvovirus* (PPV2 and PPV3), *Copiparvovirus* (PPV4–PPV6), and the unassigned genus *Chapparvovirus* (PPV7) [7–9]. PPV3, also previously referred to as porcine hokovirus (PhoV), belongs to the ungulate *Tetraparvovirus 2* species and is presently grouped together with PPV2 in the genus *Tetraparvovirus*, a recently discovered swine parvovirus that is closely related to human parvovirus 4/5 and that was first described in Hong Kong [10, 11]. In addition, PBoV is an ssDNA virus belonging to the genus *Bocaparvovirus* of the family *Parvoviridae*. It was discovered along with porcine circovirus 2 (PCV2) and TTV in the lymph nodes of pigs suffering from postweaning multisystemic wasting syndrome in Sweden in 2009 [12]. To date, three distinct swine TTV genogroups (TTV1, TTV2, and TTV3) have been discovered in domestic pigs and wild Suidae [13–15]. Although the role of individual viral pathogens in multiple infections and their participation in the development of diseases must be systematically studied, studies on the co-existence of these different DNA viruses in the same pig have been limited. In the literature, viral-viral respiratory co-infections have always had an important role in the porcine respiratory disease complex [16]. Several reports assessed the presence of two or more viral pathogens in pigs showing respiratory clinical signs in farms located in endemic regions [17, 18]. In general, co-infection is considered to lead to more severe symptoms and eventually worsen the disease outcomes because pathogen species can interact within the host. To better understand the consequences of co-infections, viral interference was progressively more frequently measured. Thus, many studies are increasingly investigating interactions between pathogens to better understand the prevalence of DNA virus co-infection in pig reservoirs worldwide.

According to the best of our knowledge, very few reports in Vietnam have looked at a range of pathogens simultaneously. These authors mainly assessed the seroprevalence of PCV2, porcine reproductive and respiratory syndrome virus (PRRSV), *Mycoplasma hyopneumoniae*, Japanese encephalitis virus, and leptospirosis, or investigated the co-infections between PCV2 and PRRSV, *M. hyopneumoniae*, and *Haemophilus parasuis* [19, 20]. It is generally accepted that the cause of a disease is based on a pattern of coinfecting pathogens rather than on individual infectious agents [21].

Therefore, this study aimed to investigate the appearance of single and multiple DNA viral infections, including PPV, PBoV, and TTV in the context of disease outbreak in Vietnamese pigs.

## Materials and Methods

### Ethical approval and Informed consent

The study protocol was reviewed and approved by the Committee on Animal Research and Ethics, Vietnam National University of Agriculture (No. TY-KHCN-NN-01, TY-KHCN-NN-02 and TY-KHCN-NN-03). Consent was provided by the relevant pig farm owners for the participation of their animals in blood collection and internal organ sampling.

### Study period and location

The study was conducted from January 2016 to December 2017 and January to December 2021. All samples from 11 provinces were collected, including three regions: Northern, Central, and Southern Vietnam.

### Sampling

This study involved samples from 11 provinces in Vietnam. A collection of 174 PCV2-positive samples (n = 86 for 2017 and n = 88 for 2021) originating from pigs showing clinical diseases of postweaning multisystemic wasting syndrome (PMWS) and PCV2-associated respiratory diseases were analyzed. Each tissue sample was subsequently homogenized and dissolved into a 10% suspension in 1× phosphate-buffered saline buffer and then stored at −70°C until its use. All samples were screened at the Laboratory of Veterinary Microbiology and Infectious Diseases, Faculty of Veterinary Medicine, Vietnam National University of Agriculture, Vietnam.

### Isolation and purification of total DNA

Total DNA from the 10% suspension was separated according to the following steps: (i) Lysis of the sample (250 μL) in sucrose/proteinase K solution (500 μL) at 56°C/90 min, (ii) phase separation of the DNA with phenol-chloroform-isoamyl solution (200 μL), (iii) precipitation of the DNA with isopropyl at −20°C/15 min, (iv) washing of the DNA precipitate with 70% alcohol, and (v) drying and dissolution of the DNA precipitate in 30 μL of TE buffer (pH 8.0). Between steps (ii) to (iv) there was a centrifugation step at 9660× g/15 min at 4°C.

### Polymerase chain reaction (PCR) strategy

A commercial PCR kit (i-StarMaster, iNtRON Biotechnology, Korea) consisting of a PCR buffer, dNTPs, MgCl_2_, and Taq DNA polymerase was used. The PCR reaction (20 μL) was mixed according to the manufacturer’s instructions. Primers used for this study are as follows: PPV1 [22], PPV2, PPV3, PPV4 [23]; TTV1 and TTV2 [24]; and PBoV [25]. The sequencing primers of the PCR reaction are shown in Table-1.

**Table-1:** Primers used in this study.

Virus	Primers	Sequences (5′-3′)	Size
PPV1	PPV1-F	AGTTAGAATAGGATGCGAGGAA	265
	PPV1-R	AGAGTCTGTTGGTGTATTTATTGG	
PPV2	PPV2-F	GCGCATTCGCCAAACTAGCTC	199
	PPV2-R	GTTTGCCCTTAATGCGATCC	
PPV3	PPV3-F	GTGGCAGTGATATTGCATCG	247
	PPV3-R	TGGCAGTCATTGAATGGAAA	
PPV4	PPV4-F	ACAAGGTGGAGGAACGTTTG	239
	PPV4-R	TTCCATGAGGGAGAGGATTG	
PBoV	PBoV-F	ACAGGCAGCCGATCACTCACTAT	680
	PBoV-R	CTCGTTCCTCCCATCAGACACTT	
TTV1	TTV1-F	CGGGTTCAGGAGGCTCAAT	305
	TTV1-R	GCCATTCGGAACTGCACTTACT	
TTV2	TTV2-F	TCATGACAGGGTTCACCGGA	252
	TTV2-R	CGTCTGCGCACTTACTTATATACTCTA	

PPV=Porcine parvovirus (e.g., PPV1, PPV2, PPV3, and PPV4), PBoV=Porcine bocavirus, TTV=Torque teno virus (TTV1 and TTV2)

## Results

### Viral DNA prevalence in the specimen by PCR

**Figure-1:**
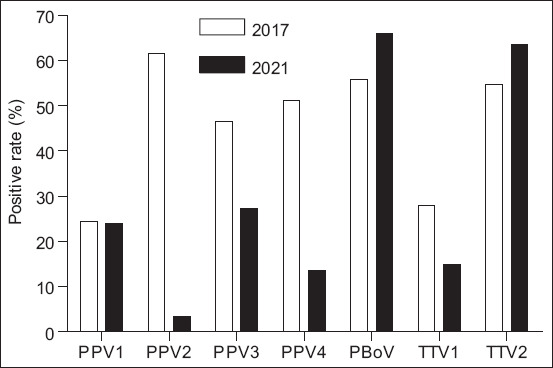
The circulation of DNA viruses in PCV2-positive samples in Vietnamese pigs in 2017 and 2021. PPV=Porcine parvovirus (e.g., PPV1, PPV2, PPV3, and PPV4), TTV=Torque teno virus (TTV1 and TTV2), PBoV=Porcine bocavirus.

To determine the current circulation of PPV (e.g., PPV1, PPV2, PPV3, and PPV4), PBoV, and TTV (TTV1 and TTV2), 174 PCV2-positive samples were examined by PCR. As expected, among four genotypes of PPV, PPV2 (61.6%) was found to be the predominant genotype in 2017 and had a detection rate of 3.4% in 2021. The detection rates of PPV3 and PPV4 were 46.5% and 51.2% in 2017 and 27.3% and 13.6% in 2021, respectively. Interestingly, the burden of PPV1 in 2021 was similar to that in 2017. In addition, two viruses that were first recorded in Vietnam, TTV and PBoV, had detection rates of 27.9% (TTV1), 57.7% (TTV2), and 55.8% (PBoV) in 2017. Unlike TTV1 (14.8%), PBoV (65.9%), and TTV2 (63.6%) were found in relatively high frequencies in 2021 (Figure-1 and Table-2).

**Table-2:** The circulation of DNA viruses in PCV2-positive samples (n = 86 for 2017 and n = 88 for 2021) in Vietnamese pigs.

Viruses	Number of PCV2-positive samples	

	Number of positives (%) in 2017	Number of positives (%) in 2021
PPV1	21 (24.4)	21 (23.9)
PPV2	53 (61.6)	3 (3.4)
PPV3	40 (46.5)	24 (27.3)
PPV4	44 (51.2)	12 (13.6)
PBoV	48 (55.8)	58 (65.9)
TTV1	24 (27.9)	13 (14.8)
TTV2	47 (54.7)	56 (63.6)

PPV=Porcine parvovirus, (e.g., PPV1, PPV2, PPV3, and PPV4), PBoV=Porcine bocavirus, TTV=Torque teno virus (TTV1 and TTV2)

### Co-infection status of DNA viruses in the PCV2-positive clinical samples

Co-infection of viruses and/or bacteria has been frequently reported in the field [26, 27]. To explore whether there were any coinfecting viruses present in our PCV2-positive samples, we investigated the presence of PPV (e.g., PPV1, PPV2, PPV3, and PPV4), PBoV, and TTV (TTV1 and TTV2) by PCR in this study. As shown in Table-3, out of the 174 PCV2-positive samples examined, 2.3% and 6.8% showed no co-infection in 2017 and 2021 in this study. Moreover, 1 (1.2%), 3 (3.5%), 2 (2.3%), and 1 (1.2%) exhibited PPV1, PPV2, PBoV, and PPV4 infection alone, respectively, in 2017, and no infection in 2021. The positive rate was observed for PBoV (2.3%, 13.6%) and TTV2 (1.2%, 10.2%) in 2017 and 2021.

**Table-3:** The prevalence of DNA virus co-infection in PCV2-positive samples (n = 86 for 2017 and n = 88 for 2021) in Vietnamese pig herds.

Co-infection combination	Number of viruses	Number of PCV2-positive samples	

		Number of positives (%) in 2017	Number of positives (%) in 2021
Negative	0	2 (2.3)	6 (6.8)
PPV1	1	1 (1.2)	0 (0)
PPV2	1	3 (3.5)	0 (0)
PPV4	1	1 (1.2)	0 (0)
PBoV	1	2 (2.3)	12 (13.6)
TTV2	1	1 (1.2)	9 (10.2)
PPV1-PPV3	2	0 (0)	1 (1.1)
PPV1-PPV4	2	0 (0)	1 (1.1)
PPV1-TTV2	2	1 (1.2)	1 (1.1)
PPV2-PPV3	2	1 (1.2)	1 (1.1)
PPV2-TTV2	2	1 (1.2)	0 (0)
PPV2-PBoV	2	2 (2.3)	1 (1.1)
PPV3-PPV4	2	1 (1.2)	0 (0)
PPV3-PBoV	2	6 (7.0)	4 (4.5)
PPV3-TTV2	2	0 (0)	3 (3.4)
PPV4-PBoV	2	2 (2.3)	1 (1.1)
PPV4-TTV2	2	1 (1.2)	1 (1.1)
PBoV-TTV1	2	0 (0)	2 (2.3)
PBoV-TTV2	2	0 (0)	9 (10.2)
TTV1-TTV2	2	1 (1.2)	1 (1.1)
PPV1-PPV4-TTV2	3	0 (0)	1 (1.1)
PPV1-PBoV-TTV2	3	0 (0)	9 (10.2)
PPV1-TTV1-TTV2	3	0 (0)	1 (1.1)
PPV1-PPV3-PBoV	3	1 (1.2)	0 (0)
PPV2-PPV3-PPV4	3	2 (2.3)	0 (0)
PPV2-PPV3-PBoV	3	2 (2.3)	0 (0)
PPV2-PBoV-TTV2	3	1 (1.2)	0 (0)
PPV2-TTV1-TTV2	3	2 (2.3)	0 (0)
PPV2-PPV4-PBoV	3	1 (1.2)	0 (0)
PPV3-PPV4-PBoV	3	4 (4.7)	0 (0)
PPV3-PBoV-TTV1	3	0 (0)	1 (1.1)
PPV3-PBoV-TTV2	3	2 (2.3)	7 (8.0)
PPV4-PBoV-TTV2	3	1 (1.2)	3 (3.4)
PPV4-PBoV-TTV1	3	1 (1.2)	0 (0)
PPV4-TTV1-TTV2	3	2 (2.3)	2 (2.3)
PBoV-TTV1-TTV2	3	0 (0)	2 (2.3)
PPV1-PPV2-PPV3-TTV2	4	1 (1.2)	0 (0)
PPV1-PPV2-PBoV-TTV2	4	1 (1.2)	0 (0)
PPV1-PPV3-PBoV-TTV1	4	0 (0)	2 (2.3)
PPV1-PPV3-PPV4-TTV2	4	0 (0)	1 (1.1)
PPV1-PPV3-PBoV-TTV2	4	0 (0)	2 (2.3)
PPV1-PPV4-PBoV-TTV2	4	0 (0)	1 (1.1)
PPV1-PPV4-TTV1-TTV2	4	0 (0)	1 (1.1)
PPV2-PPV3-PPV4-PBoV	4	4 (4.7)	0 (0)
PPV2-PPV3-PPV4-TTV2	4	2 (2.3)	0 (0)
PPV2-PPV3-PBoV-TTV2	4	3 (3.5)	1 (1.1)
PPV2-PPV3-TTV1-TTV2	4	2 (2.3)	0 (0)
PPV2-PPV4-TTV1-TTV2	4	1 (1.2)	0 (0)
PPV3-PPV4-PBoV-TTV1	4	1 (1.2)	0 (0)
PPV3-PBoV-TTV1-TTV2	4	0 (0)	1 (1.1)
PPV1-PPV2-PPV4-PBoV-TTV1	5	1 (1.2)	0 (0)
PPV1-PPV2-PPV3-TTV1-TTV2	5	1 (1.2)	0 (0)
PPV1-PPV2-PPV3-PPV4-TTV2	5	3 (3.5)	0 (0)
PPV1-PPV2-PPV3-PPV4-PBoV	5	1 (1.2)	0 (0)
PPV1-PPV2-PPV4-TTV1-TTV2	5	1 (1.2)	0 (0)
PPV1-PPV3-PPV4-TTV1-TTV2	5	2 (2.3)	0 (0)
PPV2-PPV3-PPV4-PBoV-TTV1	5	1 (1.2)	0 (0)
PPV2-PPV3-PPV4-PBoV-TTV2	5	4 (4.7)	0 (0)
PPV2-PPV3-PPV4-TTV1-TTV2	5	2 (2.3)	0 (0)
PPV2-PPV3-PBoV-TTV1-TTV2	5	3 (3.5)	0 (0)
PPV1-PPV2-PPV3-PPV4-PBoV-TTV1	6	1 (1.2)	0 (0)
PPV1-PPV2-PPV3-PPV4-PBoV-TTV2	6	3 (3.5)	0 (0)
PPV1-PPV2-PPV3-PPV4-TTV1-TTV2	6	1 (1.2)	0 (0)
PPV2-PPV3-PPV4-PBoV-TTV1-TTV2	6	1 (1.2)	0 (0)
PPV1-PPV2-PPV3-PPV4-PBoV-TTV1-TTV2	7	1 (1.2)	0 (0)

PPV=Porcine parvovirus (e.g., PPV1, PPV2, PPV3, and PPV4), TTV=Torque teno virus (TTV1 and TTV2), PBoV=Porcine bocavirus

Double infection was found in 16/86 (18.6%) animals in 2017 and 26/88 (29.5%) in 2021. Triple infection was found in 19/86 (22.1%) animals in 2017 and 26/88 (29.5%) in 2021.

**Figure-2:**
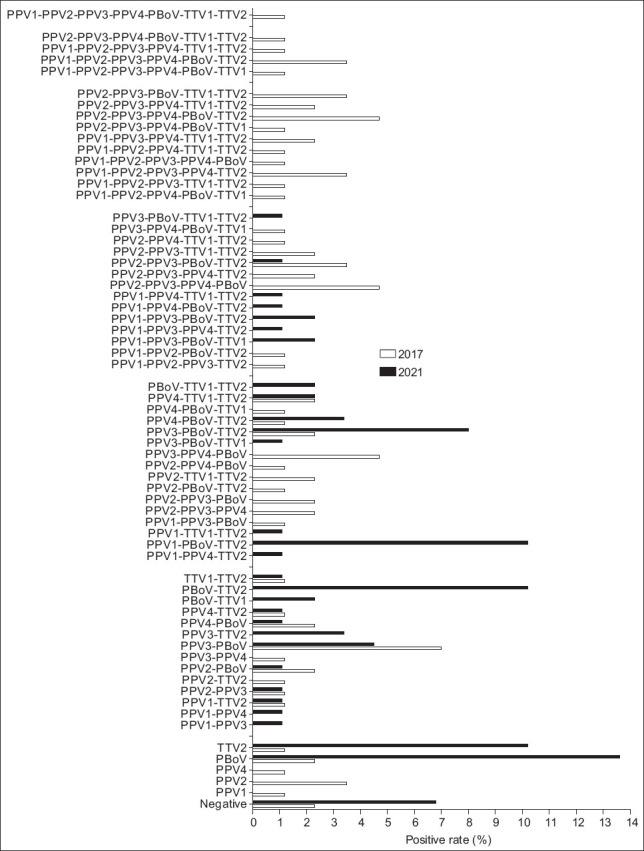
The rate of co-infection combinations of three tested pathogens in Vietnamese pigs from 11 provinces in 2017 and 2021. PPV=Porcine parvovirus (e.g., PPV1, PPV2, PPV3, and PPV4), TTV=Torque teno virus (TTV1 and TTV2), PBoV=Porcine bocavirus.

The most frequent combination of infections were PBoV-TTV2 and PPV1-PBoV-TTV2 with a rate of 9/88 (10.2%) for double and triple infections in 2021. Simultaneous detection of more than three viruses was relatively low in 2021 (Figure-2 and Table-3).

## Discussion

To the best of our knowledge, this study is the first to identify coinfection of the DNA viruses PPV, TTV, and PBoV in the context of an outbreak in Vietnamese pigs. Specifically, we showed the prevalence of a single DNA virus infection from PCV2-positive specimens. In addition, we demonstrated the simultaneous detection of multiple DNA viruses of different prevalences in 2016–2017 in Vietnamese pig herds.

In the literature, PPV is endemic in most of the world. The virus can be found in all pig herd categories, including boars and fattening pigs. Many studies have shown that PPV is widely distributed in pig herds all over the world and appears to widely vary from lower prevalences in Hungary (9.7%) [28], America (12.4%) [29]; Germany (32.7%) [30], and Portugal (24%) [10] to higher prevalences in China (44.4−51.3%) [31]. Along this line, one of the novel agents investigated in this study is TTV, a virus that was first detected in 1997 in Japan [32] and is frequently isolated from pigs with respiratory disease, immune system disorders, or liver disease. Torque teno virus is the primary cause of disease in pigs and can cross-infect between species. We found that PPV2 and TTV2 were the most prevalent (61.6% and 54.7%, respectively) viruses in 2017, with a similar finding in 2016 (Table-S1). This was consistent with the above reports, indicating that these viruses may have been the causative agents of disease in pigs in Vietnam during 2016 and 2017. Interestingly, unlike PPV2, which had a low detection rate in 2021, PPV1 generally remained constant in 2017 and 2021 despite the use of inactive PPV1 vaccines on swine farms in Vietnam. More research is needed to investigate the prevalence of PPV1 and its association with reproductive disorders of Vietnamese sows.

In the present study, PBoV had a relatively high prevalence of 65.9% in 2021. PBoV was first isolated in Sweden from lymph node samples from pigs infected with PMWS in 2009 [33] and was subsequently recorded in North America, Asia, Great Britain, Eastern Europe, and Africa [12]. Even though PBoV has been identified in many countries, its prevalence may differ based on the geographical location, age of the pigs, and pig herd management. Our finding suggested that PBoV is also widely distributed in Vietnam. To date, PBoV may not be directly associated with disease and may function as a helper virus for triggering other infectious agents [34]. Blomstrom *et al*. [35] reported that the positive rate of PBoV in pigs with PMWS was 88%, while that in healthy pigs was 46%. These reports might be explained in part by the relatively high rate of infection of PBoV viruses in this study. Consequently, the detection rate of TTV and PBoV in our study is the first identification of such co-infection in Vietnam, and the detection rate was relatively consistent with other reports.

Furthermore, some studies demonstrated that PPV was the main cause of embryo infection and fetal death [36–38]. PPV has also been proposed as a contributor to PMWS in pigs infected with PCV2 [39, 40]. One recent study also suggested that PCV2 and PPV co-infection may play an important role in PMWS in pigs [41]. Together with recently obtained global data, our findings highlight the emergence of PPV1 and PPV3 as the primary group currently affecting pig herds. In another study, there was no significant difference in the prevalence of TTV infection in pigs infected with PCV2, but co-infection with TTV could be a risk factor for pigs to have TTV symptoms [42]. In our study, the most frequent viral combinations in 2021 were PBoV-TTV2 and PPV1-PBoV-TTV2; a similar positive rate was found in 2016 with the dominant genotype being PPV2-PPV3-PPV4-TTV2 combinations (Table-S2). Thus, it is evident that the high prevalence of TTV2 alongside the high prevalences of PPV1 and PBoV hinders the understanding of the pathogenic role of TTV infection in Vietnamese pig herds.

It is well-known that PBoV is frequently involved in co-infections in pigs. One previous study identified a double infection between PBoV and PPV2 and PPV2 and PPV4 [28]. Recently, another study indicated that of 484 samples from PMWS cases, only 1.9% of samples were infected with PCV2 alone; 51.9%, 35.5%, 5.4%, and 15% of samples were also infected with PRRSV, *M. hyopneumoniae*, swine influenza virus, and PPV, respectively [43]. In addition, another report suggested that the co-infection rate of PCV2 and PPV4 was 20.2% [44]. Intriguingly, we are highly convinced that the identification of the relatively high co-infection rate of PBoV with PPV1 in this study could play a role in the outbreak of PMWS in Vietnamese pigs.

Collectively, both single and multiple viral infections were related to the age of animals [24] and were most frequently observed in pigs after weaning. Our findings of a high prevalence of DNA viruses in diseased pigs confirm that multiple infections are associated with the presence of disease in Vietnamese pig herds. It should be noted that measuring viral loads has been proposed as an important criterion to form a solid evidence base for tracking the clinical manifestations of PCV2 and PCV3 infections [45]. However, our study did not infer any significance about the variability of the virus titers between groups and identified the pathogens in tissue samples obtained from diseased and healthy pigs. Through further study, the linkage between the detected viruses and their clinical relevance should be established. Therefore, the PCR-positive clinical samples should be subjected to virus isolation and the pathogenicity of the isolated viruses should be evaluated in Vietnamese pigs.

## Conclusion

Taken together, this study provides evidence that the most important coinfecting pathogens are PPV1 and TTV2, followed by PBoV, which are involved in most cases in Vietnamese pigs. These findings not only contribute to the field at large but also highlight that the treatment of relevant co-infections seems to hold promise for improved health outcomes in Vietnamese pigs.

## Authors’ Contributions

VGN, HAD, and HTPL: Conceived, designed, and supervised the study. TTN, TMLH, and HTPL: Collected samples. BHN and LAMP: Performed the laboratory procedures. VGN, HAD, TTN, and HTPL: Analyzed the data and edited the final manuscript. All authors have read and approved the final manuscript.

## Supplementary data

**Table-S1:** The prevalence of DNA viruses in PCV2-positive samples (n = 78) in Vietnamese pigs in 2016.

Viruses	Number of positives (%) in 2016
PPV1	ND
PPV2	63 (80.8)
PPV3	39 (50.0)
PPV4	13 (16.7)
PBoV	ND
TTV1	ND
TTV2	44 (56.4)

ND=Not done, PPV=Porcine parvovirus (e.g., PPV1, PPV2, PPV3, and PPV4), TTV=Torque teno virus (TTV1 and TTV2), PBoV=Porcine bocavirus

**Table-S2:** The prevalence of co-infection of DNA viruses in PCV2-positive samples (n = 78) in Vietnamese pig herds in 2016.

Co-infection combination	Number of viruses	Number of positives (%) in 2016
Negative	0	7 (9.0)
PPV2	1	18 (23.1)
TTV2	1	5 (6.4)
PPV3-TTV2	2	2 (2.6)
PPV2-PPV3-PPV4	3	9 (11.5)
PPV2-PPV3-TTV2	3	21 (26.9)
PPV3-PPV4-TTV2	3	1 (1.3)
PPV2-PPV3-PPV4-TTV2	4	15 (19.2)

PPV=Porcine parvovirus (e.g., PPV2, PPV3, and PPV4), TTV=Torque teno virus (TTV2)
